# A preliminary study of the use of MinION sequencing to specifically detect Shiga toxin-producing *Escherichia coli* in culture swipes containing multiple serovars of this species

**DOI:** 10.1038/s41598-023-35279-1

**Published:** 2023-05-22

**Authors:** Hege S. Tunsjø, Ingvild Falkum Ullmann, Colin Charnock

**Affiliations:** 1 Department of Life Sciences and Health, Oslo Metropolitan University, Oslo, Norway; 2 Agency for Water and Wastewater Services, City of Oslo, Oslo, Norway

**Keywords:** Applied microbiology, Bacteria, Bacteriology, Biogeochemistry, Clinical microbiology, Pathogens, Microbiology, Molecular biology

## Abstract

An important challenge relating to clinical diagnostics of the foodborne pathogen Shiga toxin-producing *E. coli* (STEC), is that PCR-detection of the shiga-toxin gene (*stx*) in DNA from stool samples can be accompanied by a failure to identify an STEC isolate in pure culture on agar. In this study, we have explored the use of MinION long-read sequencing of DNA from bacterial culture swipes to detect the presence of STEC, and bioinformatic tools to characterize the STEC virulence factors. The online workflow “What’s in my pot” (WIMP) in the Epi2me cloud service, rapidly identified STEC also when it was present in culture swipes together with multiple other *E. coli* serovars, given sufficient abundance. These preliminary results provide useful information about the sensitivity of the method, which has potential to be used in clinical diagnostic of STEC, particularly in cases where a pure culture of the STEC isolate is not obtained due to the ‘STEC lost Shiga toxin’ phenomenon.

## Introduction

Shiga toxin-producing *Escherichia coli* (STEC) are foodborne pathogens capable of causing severe gastrointestinal and systemic disease^1^. STEC genomes are complex and contain many virulence genes on plasmids, bacteriophages and insertion sequences. Shiga toxin encoding genes (*stx1*/*stx2*) are located on different prophages that integrate into the *E. coli* genome, and their toxin products are essential for development of hemorrhagic colitis and hemolytic uremic syndrome^2,3^. Detection of *stx1* and/or *stx2* is, therefore, commonly used in clinical diagnostics as a marker of the pathotype of *E. coli* STEC. Primary diagnostic laboratories in Norway have switched from culture-based diagnostics to PCR-based detection of gastrointestinal pathogens directly in stool samples^4,5^. Detection of *stx1*/*stx2* in stool DNA is usually followed up by selective culture plating, and a culture swipe is assessed for toxigenic potential by the same PCR assay. Finally, detection of *stx1*/*stx2* in the culture swipe will typically lead to testing of multiple single colonies to identify the STEC isolate in pure culture^6^. However, failure to identify an STEC isolate in pure culture is not uncommon^7,8^. There are several possible reasons for failure to isolate STEC on agar from stx-positive swipes: for example, activation of *stx* expression and induction into the phage lytic cycle will result in cell lysis and low concentrations of intact STEC cells^3^. Furthermore, in the presence of a competitive microbiota (e.g., commensal *E. coli*), recovery of an STEC isolate on an agar plate may be technically challenging^9^. Additionally, *stx* may spontaneously excise from the genome without subsequent cell lysis, resulting in *E. coli* without integrated *stx*; a phenomenon which has been termed ‘STEC lost shiga toxin’—STEC-LST (10). A mix of STEC and STEC-LST colonies on agar renders the recovery of an *stx*-positive isolate difficult^10–13^. Finally, the absence of STEC growth on agar following a positive stx-PCR could be due to presence of free *stx*-phages, rather than STEC cells in the patient’s stool sample, suggesting that the patient is not in fact infected with STEC^14^.

As a guideline for choice of patient treatment and infection control measures, accurate diagnostics to identify whether a patient is infected with STEC, or simply a carrier of free *stx-*phages, is important. Furthermore, classification of STEC serotype and pathotype is paramount during an outbreak incident. Many public health agencies currently employ Next Generation Sequencing (NGS) to characterize isolates of foodborne pathogens such as STEC, in order to obtain specific information on serotype and virulence factors^15,16^. Short-read sequencing platforms like Illumina MiSeq or NextSeq are widely used, and the technology provides sequence data with high accuracy. Short read sequencing has, however, limitations with respect to the assembly of repetitive regions and regions showing high similarity in a genome that can range up to hundreds of kilobases^17^. Recently, Oxford Nanopore Technologies’ MinION small sequencing device was evaluated and found to be an accurate and economical option for whole genome sequencing of STEC isolates^18^. The technology is also well-suited for use by primary diagnostic laboratories, and the long reads obtained with MinION may be particularly useful in resolving cases with STEC-LST or samples with multiple strains. The long reads are likely to overlap and may facilitate a positive identification of the genome of interest as well as enable its characterization with respect to pathogenic potential. In this study, we have evaluated the use of long-read sequencing with MinION for the detection of STEC in culture swipes harboring different combinations of STEC and STEC-LST and other *E. coli* strains. The purpose of the investigation was to evaluate the usefulness of the method to identify the STEC serotype and pathotype present in cases where recovery of a pure STEC isolate by culture techniques is unsuccessful following a positive *stx*-PCR. This study provides a useful first set of experiments to obtain an indication of the sensitivity of the method, but is not a real substitute for data from clinical samples.

## Material and methods

### Bacterial strains

*Escherichia coli* representing four different serotypes and pathotypes were used in the experiments: Shiga toxin producing *E. coli* (STEC) O145:H28, Enteropathogenic *E. coli* (EPEC) O21, *E. coli* (EAEC) O104:H4 and an extraintestinal pathogenic *E. coli* (ExPEC) O6:H31. The O145:H28 isolate was also used as an STEC-LST variant that had lost the *stx* gene. STEC O145:H28 was previously characterized by our group^13^. Virulence genes of typical EPEC O21 and EAEC O104:H4 were determined by Clondiag GmbH using microarray technology (Clondiag GmbH, Jema, Germany).

### Preparation of bacterial inoculums and DNA extraction

**Figure 1:**
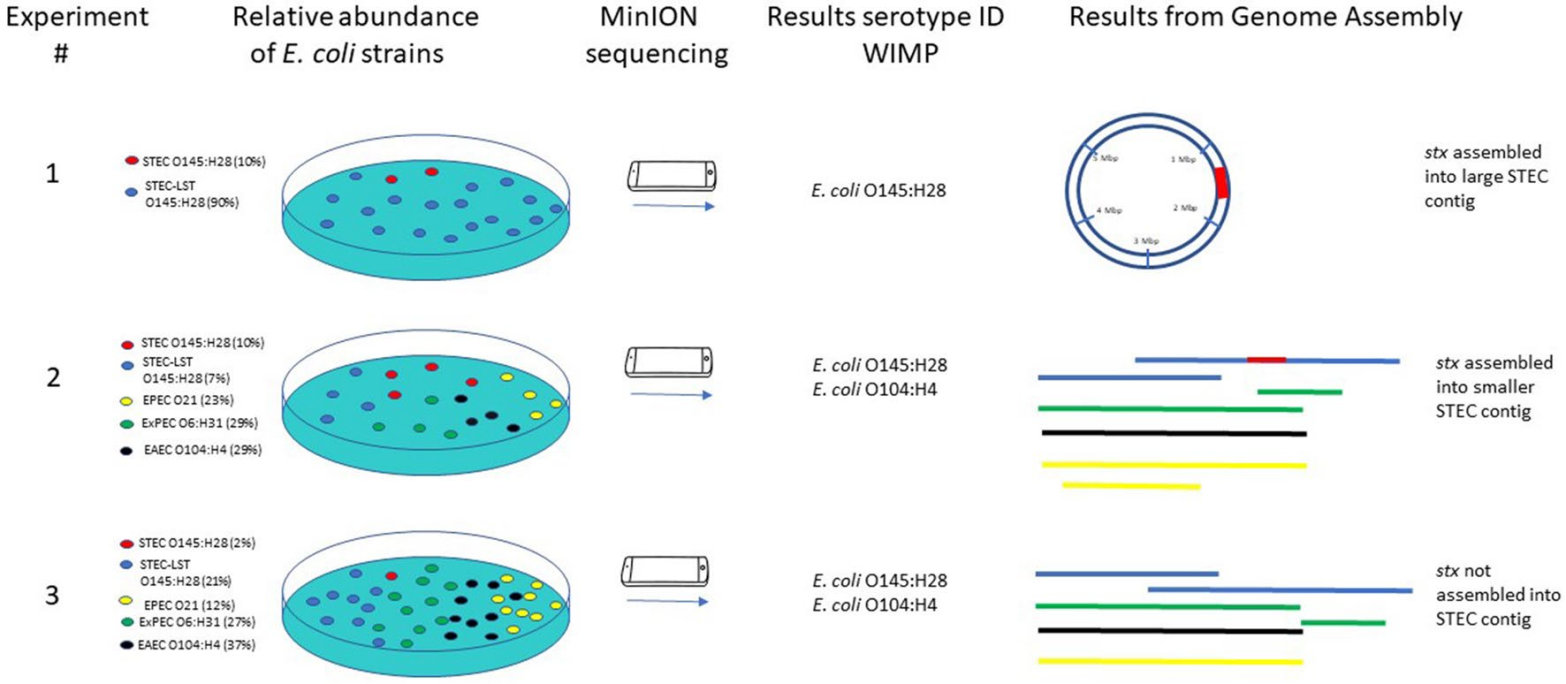
Experimental setup and summary of main results from the study. Three experiments were performed with different relative abundances of *E. coli* strains. After sequencing with MinION, the online workflow WIMP was used to identify *E. coli* strains or serotypes. Sequence reads were then assembled to confirm *stx* integration in an *E. coli* genome.

All strains were cultured on lactose agar. To mimic the diagnostic procedure under evaluation, DNA was extracted from mixed bacterial growth on agar in three different combinations as follows. In experiment 1, one loopful of bacterial colonies from STEC-LST O145:H28 and a pinpoint amount of colony material from STEC O145:H28 were suspended in PureLink® lysisbuffer (Thermo Fisher Scientific, Waltham, MA, USA) for DNA extraction. In experiment 2, one loopful of bacterial colonies from each of the following strains was suspended in lysis buffer: STEC O145:H28, STEC-LST O145:H28, EAEC O104:H4, EPEC O21 and ExPEC O6:H31. Experiment 3 was similar to experiment 2 except for that STEC O145:H28 (with *stx*) was added in a smaller amount (pinpoint of colony material). Figure 1 illustrates the experimental setup. PureLink® Genomic DNA extraction kit (Thermo Fisher Scientific) was used for DNA extraction, and DNA concentrations were measured using Qubit 4 Fluorometric quantification (ThermoFisher Scientific) employing the dsDNA Broad Range Assay according to the manufacturer’s instructions. Nanodrop™ 2000 (Thermo Fisher Scientific) was used to control for impurities (ratios 260/280 and 260/230).

### qPCR quantification

To mimic the diagnostic procedure under evaluation, DNA extraction was performed from mixed bacterial growth on agar plates, and the relative abundance of each strain was subsequently quantified with specific qPCR assays. Shiga toxin (*stx)* qPCR was used to quantify STEC^19^, while a PCR assay targeting the virulence factor *ehxA* was used as a measure for total quantities of STEC O145:H28 and STEC-LST O145:H28^20^. PCR assays targeting O21, O104 and *cnf1* were used to quantify EPEC O21, EAEC O104:H4 and ExPEC O6:H31, respectively^21–23^. TaqMan PCR-assays were performed using BrilliantIII UltraFast qPCR mix (Agilent Technologies Inc, Santa Clara, CA, USA) with the following cycling parameters: Initiation 3 min 95 °C, 40 cycles with 15 s 95 °C and 30 s 60 °C. SYBR Green PCR assays were performed using Quantifast SYBR Green PCR kit (Qiagen, Hilden, Germany) with the following cycling parameters: initiation 5 min 95 °C, 40 cycles with 10 s 95 °C, 30 s 56 °C and 30 s 72 °C. Primer and probe concentrations were 200 nM and 400 nM, respectively. Primer/probe sequences, PCR efficiencies and amplicon size for all PCR assays used in this study are listed in Table 1.

**Table 1:** Primers and probes used in the study.

*E. coli* strain	Target gene	Primer/probe sequence 5’–3’	bp*	PCR E (%)	References
STEC O145:H28	*stx2*	F: GGG CAG TTA TTT TGC TGT GGA R: GAA AGT ATT TGT TGC CGT ATT AAC GA P: ATG TCT ATC AGG CGC GTT TTG ACC ATC TT	131	96	^19^
	*ehxA*	F: GTG TCA GTA GGG AAG CGA ACA R: ATC ATG TTT TCC GCC AAT G P: CGT GAT TTT GAA TTC AGA ACC GGT GG	125	108	^20^
EPEC O21	*wzxO21*	F: CTG CTG ATG TCG CTA TTA TTG CTG R: TGA AAA AAA GGG AAA CAG AAG AGC C	209	95	^21^
EAEC O104: H4	*wzxO104*	F: TGTCGCGCAAAGAATTTCAAC R: AAAATCCTTTAAACTATACGCCC P: TTGGTTTTTTTGTATTAGCAATAAGTGGTGTC	100	102	^22^
ExPEC O6:H31	*cnf1*	F: AGCGTGCAATCTATCCGTATTT R: TGGAATTTCCCCAGTATAGGTG	173	89	^23^

*bp: amplicon size in base pairs, PCR E (%): PCR efficiency. F: forward primer, R: reverse primer, P: probe.

Analytical sensitivity and PCR efficiency tests were performed for each PCR assay, using tenfold serial dilutions of DNA from pure bacterial cultures containing from 4 ng/µl to 0.04 pg/µl. Standard curves were constructed and used for PCR efficiency calculations and to quantify the different *E. coli* in the samples. Specificity tests were carried out by cross-testing the different *E. coli* isolates in each of the strain-specific PCR assays.

### MinION whole genome sequencing

Whole genome sequencing of DNA from the different *E. coli* combinations was performed using a minION MK1b device and the Rapid Barcoding Sequencing Kit (SQK-RBK004) (Oxford Nanopore Technologies, Oxford, GB), according to the manufacturer’s protocol. For each of the three experiments, approximately 400 ng DNA was loaded onto a R9.4 MinION Flow Cell (FLO-MIN107). The sequencing run was performed through the minKNOW platform using the MIN107 SQK-RBK004 protocol. The run time was 24 h.

### Processing of sequence data

Basecalling of minION sequencing data was performed using Albacore version 1.2.4 (https://github.com/Albacore/albacore). FASTQ sequences were uploaded to the Epi2me cloud service and analyzed with the workflow “What’s in my pot” (WIMP) (Epi2me, Oxford nanopore technologies) which uses the RefSeq sequence database at NCBI (https://www.ncbi.nlm.nih.gov/refseq/) for identification^24^. PoreChop version 0.2.3 (https://github.com/rrwick/Porechop) was used for adapter trimming of the sequence reads. De novo assembly was performed with Canu version 1.6^25^ and quality reports for assembled contigs were generated using QUAST^26^.

### In silico detection of virulence genes, phages, pathogenic potential, and serotyping

FASTA-files with assembled contigs from each sequencing run were submitted to the Centre for Genomic Epidemiology (CGE) (http://www.genomicepidemiology.org). The web-based tools VirulenceFinder 1.5^27^ and PathogenFinder^28^ were used to identify virulence genes and assess the pathogenic potential of strains. Sequences were also submitted to the typing services SerotypeFinder^29^ and MLSTFinder^30^. The threshold used for ID was 90%, and minimum length of overlap was set to 60%.

## Results

### Relative abundances of *E. coli* strains in different experiments

Three experiments with different combinations of each *E. coli* strain were performed; these are summarized in Fig. 1. After DNA extraction, quantification of each *E. coli* strain was performed using strain-specific qPCR assays. In experiment 2, each *E. coli* was present in similar quantities, while in experiments 1 and 3, the relative quantities of STEC O145:H28 were respectively 10 and 50 times lower than that of the other *E. coli* strains (Fig. 1). No cross-reactions were observed for any of the qPCR assays and PCR efficiencies were between 89 and 108% (Tables S1 and S2).

### MinION sequencing and Epi2me real-time data analysis

The MinION sequencing platform provided between 2.3 and 2.5 Gbp data output and sequence reads with average read lengths of 10 kB in all three experiments. The average quality score for the sequencing reads was 10. The WIMP workflow in the Epi2me cloud service correctly identified *E. coli* O145:H28 in experiment 1. *E. coli* O145:H28 was the only serotype present in this experiment as a combination of STEC and STEC-LST (ie, with and without the *stx* gene). In experiments 2 and 3, with four different *E. coli* serotypes, WIMP identified two of them, namely *E. coli* O104:H4 and *E. coli* O45:H28. Neither *E. coli* O6:H31 nor *E. coli* serotype O21 were identified by WIMP in any of these two experiments. Results are presented in Table 2.

**Table 2:** Overview of MinION sequencing data and results from bioinformatic analysis.

	Experiment 1	Experiment 2	Experiment 3
*E. coli* strains in each experiment (relative abundance)	STEC O145:H28 (~ 10%) + STEC-LST O145:H28 (~ 90%)	STEC O145:H28 (~ 10%) STEC-LST O145:H28 ~ 7%) EPEC O21 (~ 23%) EAEC O104:H4 (~ 29%) ExPEC O6:H31 (~ 29%)	STEC O145:H28 (~ 2%) STEC-LST O145:H28 (~ 21%) EPEC O21 (~ 12%) EAEC O104:H4 (~ 37%) ExPEC O6:H31 (~ 27%)
Data output minION	2.5 gB	2.3gB	2.3 gB
# of reads minION	417 000	379 000	386 920
Serotypes identified by WIMP*	O145:H28	O145:H28, O104:H4	O145:H28, O104:H4,
# of contigs after assembly CANU	3 (5,4 Mb, 141 kb, 142 kb)	135	160
PathogenFinder	Input organism predicted as human pathogen	Input organism predicted as human pathogen	Input organism predicted as human pathogen
Serogroups identified by SerotypeFinder	O145, H28	O6, H28, H4, H31	O21, H28, H4
Virulence genes identified by VirulenceFinder	STEC: stx2A, stx2B, astA, iha, ehxA, chu, cib, cif, etpD, gad, iuc, iutA, neuC, tccP, terC, traT EPEC/STEC: Eae, tir, efa1, espA/B/F/, nleA/B/C	STEC: stx2A, stx2B, astA, iha, ehxA, chu, cib, cif, etpD, gad, iuc, iutA, tccP, terC, traT EPEC/STEC: Eae, tir, efa1, espA/B/F/, nleA/B/C EAEC: aap, aar, aatA, aagA, aagB/C/D/R, aaiC, pic, capU, sepA, sigA, mcmA, safD, safe, sfaF, vat ExPEC: cnf1, usp, clbB, kpsMII, kpsE, fyuA, irp2, papA/C, hra, iss	STEC: astA, iha, ehxA, chu, cib, cif, etpD, gad, iuc, iutA, tccP, terC, traT EPEC/STEC: Eae, tir, efa1, espA/B/F/, nleA/B/C EAEC: aaiC, pic, capU, sepA, mchB/C/F, mcmA, safD, safe, sfaF, vat ExPEC: cnf1, usp, clbB, kpsE, fyuA, irp2, papA/C, hra, iss
MLSTFinder	ST6130	No ST	No ST
Plasmid Finder	RM13516 (IncFIB)	None	None

### De novo sequence assembly

In experiment 1, Canu^25^ assembled the sequencing reads into one single large contig similar in size to an *E. coli* genome (5.4 Mbp), accompanied by two smaller contigs of 141,573 and 142,342 nucleotides. In experiments 2 and 3, with several different *E. coli* strains, the sequencing reads were assembled into 135 and 160 contigs, respectively. QUAST^26^ showed that the largest contig in experiment 2 was 573,742 nucleotides and that 128 contigs were larger than 50,000 nucleotides. In experiment 3, the largest contig was 535,013 nucleotides and 125 contigs were larger than 50,000 (Table 2). All sequence data have been made available through Figshare https://doi.org/10.6084/m9.figshare.21342453.

### Detection of virulence genes and pathogenic potential

The sequence assemblies from Canu^25^ were used to assess the pathogenic potential of the strains. PathogenFinder^28^ predicted that the input organism(s) were human pathogens in all the experiments. VirulenceFinder^29^ detected important STEC virulence genes in experiment 1, for example Shiga-toxin encoding gene (*stx),* Enterohaemolysin gene *(ehxA)*, Adherence-conferring molecule gene (*iha)* and Tir-cytoskeleton coupling protein gene *(tccP)* (Table 2). For the two experiments with several different *E. coli,* the program detected multiple virulence genes associated with STEC, EAEC, EPEC and ExPEC (Table 2). However, in experiment 3 *stx* was not identified in any of the contigs after de novo assembly. Unassembled FASTQ files from experiment 3 were also submitted to VirulenceFinder, but the *stx* gene was not identified in any of the reads. Results from VirulenceFinder and PathogenFinder are summarized in Table 2.

### In silico serotyping and MLST analysis

The assembled contigs were also analysed with SerotypeFinder^28^ and MLSTFinder^30^. These are programs designed to determine the O-and H serotype and MLST-types of single strains. The SerotypeFinder only identified one O-gene in each experiment but indicated several H-genes. MLSTFinder correctly identified the MLST type of STEC in experiment 1. Results from SerotypeFinder, MLSTFinder and PlasmidFinder are summarized in Table 2.

### A protocol to identify STEC in culture swipes

**Figure 2:**
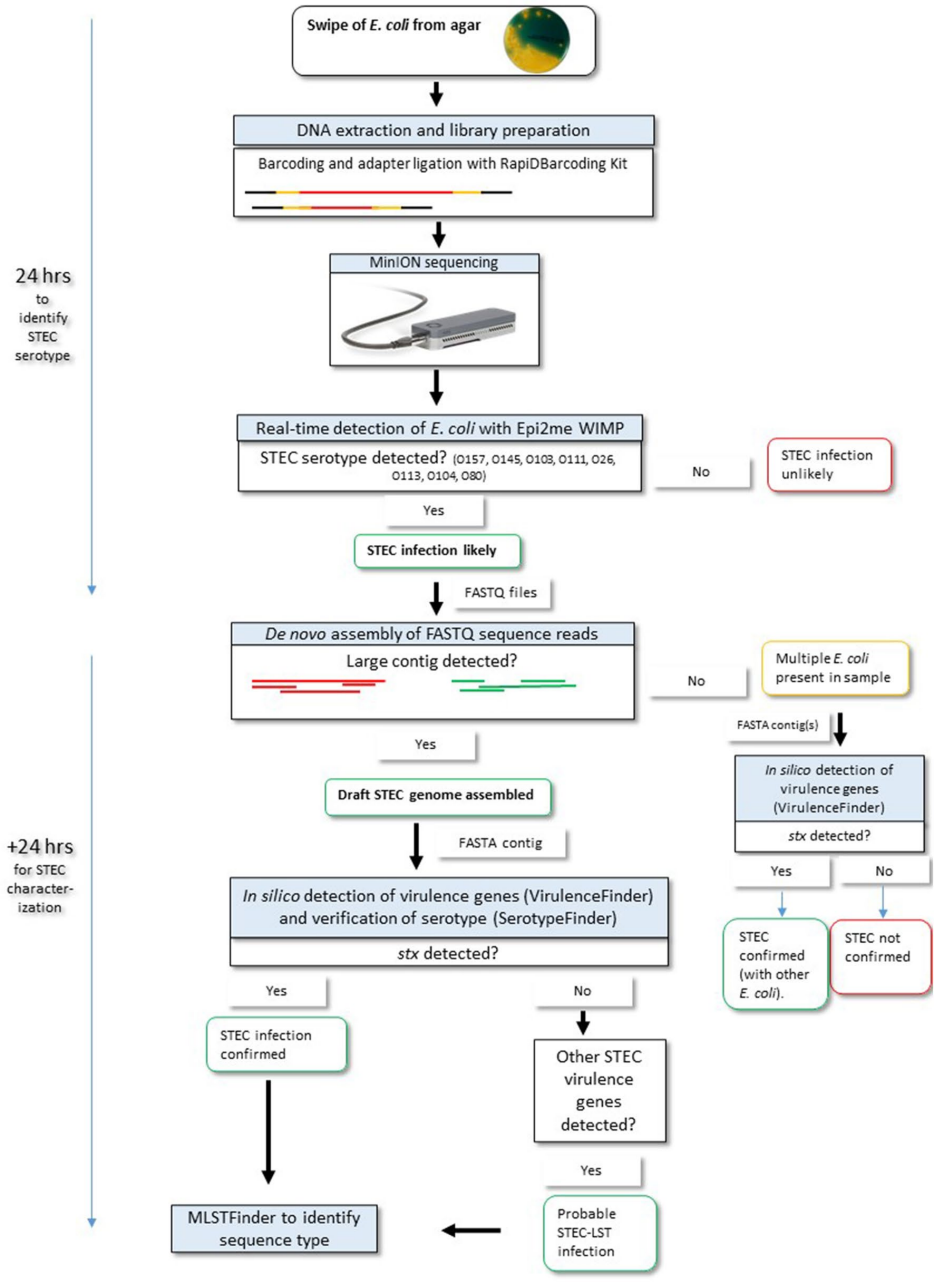
Suggested protocol for clinical diagnostics of STEC with minION sequencing of DNA extracted from culture swipes. Real-time integrated basecalling allows for direct upload of sequence reads to the Epi2me cloud service and WIMP workflow. The suggested procedure results in identification of STEC serotype in less than 24 h. For further characterization of the STEC genome, FASTQ files can be assembled and online tools such as VirulenceFinder will identify virulence factors. If a near complete genome can be assembled and *stx* is detected in it, the presence of STEC is confirmed. If de novo assembly results in multiple smaller contigs, indicative of multiple *E. coli*, *stx* may still be identified as described. However, sequence typing and detailed characterization of the STEC isolate will be difficult.

Based on the results, a protocol was suggested to improve diagnostics of STEC in samples which are *stx* PCR-postive and culture negative (Fig. 2).

## Discussion

Several studies have reported challenges associated with the confirmation of *stx* PCR-positive stool samples. Subsequent culture of the *stx*-positive strain is advised for verification of the PCR-result and for strain characterization and infection control measures. When an STEC isolate cannot be cultured, the situation is described as the ‘STEC PCR-positive/culture-negative phenomenon’^6,7^. To identify the STEC isolate, several rounds of selective plating may be necessary. The repeated plating passages may lead to induction of the phage lytic cycle, or to spontaneous excision of the prophage from the STEC genome without subsequent cell lysis, resulting in STEC-LST. Consequently, the number of STEC with intact prophages will be limited, and, therefore, difficult to detect by culture and *stx* PCR-testing of single colonies. Loss of *stx* during laboratory work is a well-known phenomenon^10–13^.

This study investigated the use of MinION sequencing technology and the proposed protocol to identify STEC in bacterial culture swipes, in cases where recovery of a pure STEC isolate is unsuccessful. When DNA from a swipe of STEC and STEC-LST was sequenced, the Epi2me workflow WIMP rapidly identified the well-known STEC serotype O145:H28. De novo assembly resulted in one large genome-sized contig, in which all STEC virulence factors were identified by user-friendly online tools from CGE, even though only 10% of the *E. coli* in the sample were STEC with intact *stx.* Additionally, SerotypeFinder identified the correct serotype and MLSTFinder identified the correct multi-locus sequence type, thus illustrating the usefulness of the proposed protocol in samples with STEC and STEC-LST.

The protocol was further evaluated with culture swipes with different *E. coli* serotypes. In these experiments, WIMP rapidly identified the two *E. coli* that may cause severe gastrointestinal disease: STEC serotype O145:H28 and EAEC serotype O104:H4. *E. coli* serotype O6:H31 and EPEC O2 were not identified by WIMP, most likely because no O6:H31 genomes and only three O2 genomes are defined at assembly level as “complete” in the NCBI RefSeq database, which is used by WIMP^24,31^. De novo sequence assembly resulted in more than 100 sequence contigs, indicating the presence of more than one *E. coli* strain*.* In experiment 2, with similar abundances of four different *E. coli* strains, several STEC virulence genes (*stx* and others) were identified by VirulenceFinder^27^, confirming STEC in the sample. Due to the presence of more than one *E. coli* strain in the sample, no result from MLSTFinder was obtained, and, therefore, genotyping of the STEC could not be achieved. Nevertheless, the approach can be used to confirm the presence of STEC in mixed *E. coli* populations. In experiment 3, where STEC was present as approximately 2% of total *E. coli*, the *stx* gene was not detected by VirulenceFinder. Based on the MinION sequencing output (2.5 Gb) for this sample, which contained four different *E. coli* genomes (estimated total genome size > 20 Mb), a theoretical sequencing coverage of approximately 100 should be expected. The *stx* gene was present in significantly lower quantities (2%) than that of the other DNA-sequences, and it is, therefore, likely that the *stx* sequence reads could have been omitted in the de novo assembly process, or simply not sequenced at all. This suggests that when the abundance of STEC is significantly lower than that of non-STEC *E. coli*, the proposed procedure will not positively confirm STEC. Nevertheless, with detection of a well-known STEC serotype, the presence of STEC-LST should be considered.

In this study, we have evaluated the usefulness of MinION whole genome sequencing of *E. coli* from culture swipes for the purpose of confirming STEC. The results provide information about the sensitivity of the method, which potentially could be useful in cases where a STEC-isolate is not obtained following a positive *stx*-PCR. The protocol could potentially also be applicable to resolve other diagnostic challenges. For example, to differentiate the gastrointestinal pathogens *Shigella* spp. and enteroinvasive *E. coli* (EIEC). The invasion plasmid antigen H gene (*ipaH),* present in both these pathogens, is often used as a qPCR target. Culture and identification of single colonies is, therefore, necessary for identification, but is not always possible^32^. When neither *Shigella* nor EIEC can be retrieved after culture of an *ipaH* PCR-positive stool sample, the proposed protocol using MinION sequencing could be considered.

A concern with the MinION nanopore sequencing technology is its higher error rate when compared to short-read sequencing technologies. This problem was highlighted by Gonzales-Escalona et al.^18^, who compared *E. coli* sequencing results using three different technologies and found that data from MinION contained several artificial indels. Still, the authors successfully characterized the STEC isolates with respect to virulence genes, plasmids, and antibiotic resistance genes, results also supported by Taylor et al.^33^. Different programs and algorithms have been presented to improve the quality of MinION data and to enable detailed SNP phylogenetic analyses^34^. Additional bioinformatic analysis could be considered if the purpose is beyond presence/absence analysis of specific genes. This was not the purpose of the present study and was therefore not performed. Recently, Maguire et al.^35^ demonstrated that complete STEC genomes could be identified in spiked water samples using a metagenomic approach and the MinION technology. Results from the present study support previous reports of the usefulness of MinION sequencing and illustrate that the technology may also be useful for STEC identification in culture swipes with multiple strains. A limitation of the present work is that the protocol described has been developed and validated using only a limited number of strains and serovars. The workflow should in later work be tested against a larger panel of especially clinical isolates and starting with fecal material naturally containing or spiked with STEC.

## Conclusion

In conclusion, the results from this study provide preliminary data about the use of MinION sequencing technology supported by user-friendly online web-tools to identify STEC in mixed samples. The protocol could be particularly useful in cases of PCR-positive/culture-negative samples caused by loss of *stx*, and it is possible that detailed characterization of the STEC genome can be obtained in samples containing both STEC and STEC-LST. The procedure is easy to implement in routine diagnostic laboratories and the instrumentation and consumables are affordable. However, more comprehensive analyses with different STEC serotypes are required to confirm the results from this study. Furthermore, the data presented are not a real substitute for data from clinical samples, and the protocol requires further testing using clinical samples to evaluate its true potential in clinical diagnostics.

## Supplementary Information

Supplementary Tables.

## Data Availability

All sequence data have been made available through Figshare. https://doi.org/10.6084/m9.figshare.21342453 or https://figshare.com/articles/dataset/Genome_assembly_of_E_coli_strain_mixes/21342453.
