# Supplementary material for: A preliminary study of the use of MinION sequencing to specifically detect Shiga toxin-producing Escherichia coli in culture swipes containing multiple serovars of this species
Source: Sci Rep. 2023 May 22;13:8239. doi: 10.1038/s41598-023-35279-1 (PMC10202931; doi:10.1038/s41598-023-35279-1)
Supplement: Supplementary file 1 — Supplementary Tables. [file 41598_2023_35279_MOESM1_ESM.docx]

Supplementary information

**A preliminary study of the use of MinION sequencing to specifically detect Shiga toxin-producing *Escherichia* *coli* in culture swipes containing multiple serovars of this species**

**Scientific Reports**

Hege S Tunsjø^1*^, Ingvild Falkum Ullmann^2^, Colin Charnock^1^

^1^Department of Life Sciences and Health, Oslo Metropolitan University, Oslo, Norway

^2^City of Oslo, Agency for Water and Wastewater services, Oslo, Norway

*Corresponding author: [hetu@oslomet.no](mailto:hetu@oslomet.no). Tel. +4795052752

Table S1. Results from qPCR quantitation of each *E. coli* strain in different experiments

|  | *stx2** | *ehxA** | *vzxO104*** | *vzxO21**** | *cnf1***** |
| --- | --- | --- | --- | --- | --- |
| Std 1 | 4 ng/µl | 4 ng/µl | 4 ng/µl | 4 ng/µl | 4 ng/µl |
| Std 2 | 4*10e-1 ng/µl | 4*10e-1 ng/µl | 4*10e-1 ng/µl | 4*10e-1 ng/µl | 4*10e-1 ng/µl |
| Std 3 | 4*10e-2 ng/µl | 4*10e-2 ng/µl | 4*10e-2 ng/µl | 4*10e-2 ng/µl | 4*10e-2 ng/µl |
| Std 4 | 4*10e-3 ng/µl | 4*10e-3 ng/µl | 4*10e-3 ng/µl | 4*10e-3 ng/µl | 4*10e-3 ng/µl |
| Std 5 | 4*10e-4 ng/µl | 4*10e-4 ng/µl | 4*10e-4 ng/µl | 4*10e-4 ng/µl | 4*10e-4 ng/µl |
| Std 6 | 4*10e-5 ng/µl | 4*10e-5 ng/µl | 4*10e-5 ng/µl | 4*10e-5 ng/µl | 4*10e-5 ng/µl |
| Neg ctrl | 0 | 0 | 0 | 0 | 0 |
| Exp. # 1 | 0,013 ± 0,004 ng/µl | 0,122 ± 0,002 ng/µl | 0 | 0 | 0 |
| Exp. # 2 | 0,182 ± 0,015 ng/µl | 0,300 ± 0,045 ng/µl | 0,534 ± 0,111 ng/µl | 0,424 ± 0,051 ng/µl | 0,526 ± 0,013 ng/µl |
| Exp. # 3 | 0,018 ± 0,009 ng/µl | 0,201 ± 0,005 ng/µl | 0,316 ± 0,112 ng/µl | 0,103 ± 0,019 ng/µl | 0,236 ± 0,022 ng/µl |

10x dilution series of different *E. coli* strains were used to generate standard curves. *STEC O145:H28, ** EAEC O104:H4, *** EPEC O21, **** ExPEC O6:H31. All values are given as mean of triplicates or parallels. Exp. = Experiment.

Table S2. Relative abundance calculation of each *E. coli* strain in different experiments. Due to varying efficiencies of the PCR assays the results are given as approximate values.

| Experiment 1 | | Experiment 2 | | Experiment 3 | |
| --- | --- | --- | --- | --- | --- |
| Quantification from qPCR | Relative abundance | Quantification from qPCR | Relative abundance | Quantification from qPCR | Relative abundance |
|  |  |  |  |  |  |
|  |  |  |  |  |  |
| *ehxA*  0,122 ng/µl  STEC and STEC-LST | 100%* | *ehxA*  0,300 ng/µl  STEC and STEC-LST | ~17%* | *ehxA*  0,201 ng/µl *STEC and STEC-LST* | ~23%* |
| *stx2*  0,013 ng/µl  STEC | ~10% | *stx2*  0,182 ng/µl  STEC | ~10% | *stx2*  0,018 ng/µl  STEC | ~2% |
| 0,122-0,013 = 109  STEC-LST | ~90% | 0,30-0,182 = 0,118  STEC-LST | ~7% | 0,201-0,018 = 0,183  STEC-LST | ~21% |
|  |  | *O104*  0,534 ng/µl  *EAEC* | 29% | *O104*  0,316 ng/µl  EAEC | ~37% |
|  |  | *O21*  0,42 ng/µl  *EPEC* | 23% | *O21*  0,103 ng/µl  *EPEC* | ~12% |
|  |  | *cnf1*  0,526 ng/µl  *ExPEC* | ~29% | *cnf1*  0,236 ng/µl  *ExPEC* | 27% |

*Quantities of STEC and STEC-LST combined
